# Matrix-based project dataset parsers

**DOI:** 10.1016/j.mex.2024.102821

**Published:** 2024-07-09

**Authors:** Zsolt T. Kosztyán, Gergely L. Novák

**Affiliations:** a Department of Quantitative Methods, University of Pannonia, Egyetem str. 10, Veszprém, H-8200, Hungary; b Continental Automotive Hungary Ltd., Házgyári str. 6-8., Veszprém, H-8200, Hungary

**Keywords:** Matrix-based projects, Structural flexibility, Project parser, Project database parser

## Abstract

There are several existing project datasets, which involve separate data sources for simulated and real projects, individual and multiprojects, and single- and multimodal attributes. In addition, their file structures are heterogeneous; therefore, scholars can usually use only one dataset to test a proposed scheduling or resource allocation algorithm. Since the internal structures of these projects are also very different, it is difficult to ensure that an algorithm optimized for a given type of project will also perform well on projects with other structures. The proposed parsing method supports researchers in:

• reading several types of projects: simulated, real, individual, and multiprojects, as well as single- and multimodal attributes;
• considering the priorities of activities and the flexibility of their dependencies, which is essential for modeling the structural flexibility employed by agile, hybrid, and extreme project management approaches;
• building a large project database for testing and comparing different scheduling and resource allocation algorithms.

## Graphical abstract

**Figure:**
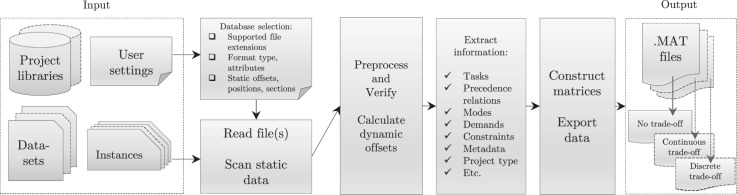

Specifications table

**Table:** 

Subject area:	*Engineering*
More specific subject area:	Operations research
Name of your method:	Project database parser
Name and reference of original method:	*N/A*
Resource availability:	https://github.com/novakge/project-parsers https://codeocean.com/capsule/9344928/tree/v1

## Background

Projects of all types can contribute almost 20 % of a country's GDP [1,7]. This is why there is much interest in scheduling projects, multiprojects, and programs. Algorithms are being developed that can solve increasingly large problems more quickly while considering even more properties of projects. It is now standard to accept a scheduling or resource allocation method only after testing the proposed algorithms on standard databases and comparing them with other recently published algorithms [8]. Researchers have been providing project databases for nearly fifty years (see, e.g. [17]), and they are still some of the most highly referenced publications to date [10]. Most of them are based on simulated databases, such as Patterson [17], SMCP and SMFF [10], PSPLIB [19], RG300 and RG30 [[6], [22]], Boctor [3], and MMLIB [18]. Additionally, there is already a database of real-life projects by Batselier and Vanhoucke [2].

Existing project databases can be grouped in several ways. Most databases contain individual project structures (see, e.g., [[10], [17], [19]]), but there are also databases with multiple projects, (see, e.g., [9]) that may run in parallel. Most project databases are based on simulated project structures (see, e.g., [[3], [18]]). The simulated project structures can support more than one completion mode. The different completion modes specify a set of alternative demands to execute a task, such as duration, cost, and resource requirements. While simulated data usually do not include cost-related information, they include various completion modes for individual and multiple project instances. The currently available database of real projects [2] incorporates cost-related information; however, it is limited to individual projects and provides a single completion mode.

Table 1 summarizes the properties of the project databases employed.

**Table 1:** Applied project databases.

Name	Project Plan	Completion Modes	Projects	Demands	Cited as
Patterson	Generated	Single	Single	Time, renewable resources	Patterson [17]
PSPLIB	Generated	Single, Multi	Single	Time, renewable/nonrenewable resources	Sprecher and Kolisch [19]
RG30, RG300	Generated	Single	Single	Time, renewable resources	Vanhoucke et al. [22]
SMCP, SMFF	Generated	Single	Single	Time, renewable resources	Kolisch et al. [10]
Boctor	Generated	Multi	Single	Time, renewable resources	Boctor [3]
MMLIB	Generated	Multi	Single	Time, renewable/nonrenewable resources	Peteghem and Vanhoucke [18]
Real-life	Collected	Single	Single	Time, cost, renewable resources	Batselier and Vanhoucke [2]
MPSPLIB	Generated	Single	Multiple	Time, renewable resources	Homberger [9]
BY	Generated	Single	Multiple	Time, cost, renewable resources	Browning and Yassine [4]
RCMPSPLIB	Generated	Single	Multiple	Time, renewable resources	Vázquez et al. [23]
MPLIB1, MPLIB2	Generated	Single	Multiple	Time, renewable resources	Van Eynde and Vanhoucke [20]

All the file structures, the presented project structures, and the features of the projects, such as task and project demands, are very different. In addition, several studied features, such as quality and flexibility, are not stored in these databases.

### Method details

**Fig. 1:**
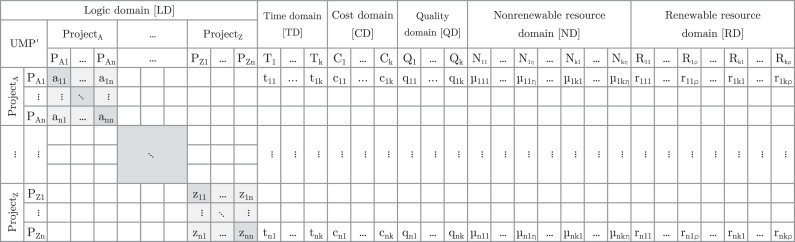
Structure of the unified matrix-based project planning model (UMP).

To overcome the limitations of existing databases, Kosztyan et al. [15] proposed a Unified Matrix-Based Project-Planning model (UMP). The UMP uses a sparse matrix to represent a single project or multiple projects. Fig. 1 shows the proposed UMP sparse matrix model.

A UMP is a generalized version of a Domain Mapping Matrix (DMM), which is used to describe a complex project [5]. The UMP specifies two mandatory domains, such as Logic Domain (LD) and Time Domain (TD) (i.e., submatrices). In addition, the UMP specifies four supplementary domains, such as Cost Domain (CD), Quality Domain (QD), Nonrenewable Resource Domain (ND), and Renewable Resource Domain (RD).

LD is binary if there is no flexible dependency between tasks and every task must be completed. However, values between 0 and 1 on the diagonal indicate supplementary tasks, and values between 0 and 1 on the out-diagonal indicate flexible dependencies. Although the parsed project databases do not contain quality or flexibility data, the addition of these submatrices (domains) already enables the modeling of these problem types.

Table 2 summarizes which kinds of project scheduling and resource allocation algorithms can be tested after the proposed matrix-based project dataset parser collects the project structures.

**Table 2:** Supported project scheduling resource allocation problems. (Note: * Quality parameters are not incorporated in the original project databases).

Abbrev.	Problem
CTCTP	Continuous Time-Cost Trade-Off Problem
DTCTP	Discrete Time-Cost Trade-Off Problem
CTQCTP	Continuous Time-Quality-Cost Trade-Off Problem
DTQCTP	Discrete Time-Quality-Cost Trade-Off Problem *
RC—CTCTP	Resource-Constrained Continuous Time-Cost Trade-Off Problem
RC-DTCTP	Resource-Constrained Discrete Time-Cost Trade-off Problem
RC—CTQCTP	Resource-Constrained Continuous Time-Quality-Cost Trade-Off Problem *
RC-DTQCTP	Resource-Constrained Discrete Time-Quality-Cost Trade-Off Problem *
MRCPSP	Multimode Resource-Constrained Project Scheduling Problem
MRCPSP-Q	Multimode Resource-Constrained Project Scheduling Problem with Quality Parameters *

The parsed project databases do not contain quality and flexibilitye parameters. If they must be included in an optimization problem, the parameters have to be specified after parsing the databases. Importantly, the problems in Table 2 are extensively researched. For fixed structures, many scheduling algorithms use different approaches to solve problems [8]. However, thus far, only a few datasets have been used to investigate the performance of the methods, so it is not possible to know which approach is the fastest for obtaining an optimal or near-optimal solution. The database built with the proposed parser supports these comparisons. In addition, all these problem solvers, which also work on flexible structures, were implemented in MATLAB by Kosztyan [13].

The parser [16] is freely available and can be downloaded from the Code Ocean site. The parser is implemented in MATLAB. The outputs are the parsed MAT files of the original datasets. The MAT files can easily be imported in Python using the SciPy library [24], for this, we also provided a working example in the parser's repository.

The first concept of matrix-based flexible project planning is based on the work of Kosztyan [11]. It is generalized to handle multiple completion modes [14] and multiproject structures [12]. The UMP is the latest version of the proposed flexible matrix-based project planning matrix published by [15].

### Method description

**Fig. 2:**
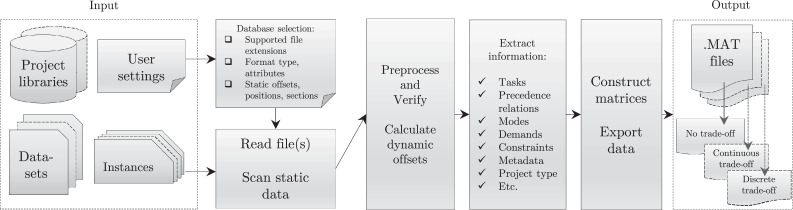
Main functionalities and operation of the parser tool.

The project parser tool reads and processes project data and exports them to a matrix-based format. Fig. 2 shows the main operation of the parser. The user selects the database to be processed (user setting), which gives the supported extension filtering the files to read.

Processing continues using the specified format and the corresponding attributes. Possible errors are handled during the parsing operation, such as missing or invalid data that might not match the expected format.

Static information and metadata are processed first, such as the number of activities and resource types, which are typically provided in the header of each instance. The data are scanned for reference lines and offsets, based on keywords or other unique identifiers. This information is also used to calculate the necessary positions and varying sizes of dynamic sections of the content.

First the logic network, then the demands and constraints are extracted. According to the selected simulation (trade-off) problem type, these are converted into project domain matrices (PDMs). After this, the specific instances can be constructed and finally, exported as MAT files.

The software has a modular architecture that provides easy maintenance for heterogeneous database formats. The architecture consists of separate files for each library (format group) supporting these functionalities. These files contain dedicated functions capable of interpreting a given format or a set of closely related or similar formats.

MATLAB's built-in functions were favored in the course of development, such as the ’textscan’ function, which is used for most data import processes with the necessary parameters, such as handling delimiters, format specifications, and variable types, and provides good performance, generic exceptions, and error handling.

Data are processed into the appropriate data structures, such as cell arrays. After this, conversion to a matrix-based format is performed from the variables, with a logic appropriate to the attributes and format.

The project parser tool has two main functionalities: reading and processing project data and exporting data to matrix-based formats.

Separate functions are provided to read and process project instances in the ’parse 〈library format〉.m’ files. To export a given dataset in a matrix-based format with the desired simulation type, ’save instance.m’ is provided with an argument for the data and its format (group). This creates the output folder, calls the associated parser function for the given format, and is called by further batch scripts to save a set of project data. To save an existing project library with all its datasets in batch mode from the data folder, ’save all 〈library〉.m’ is provided. To batch-save all libraries, datasets, and instances, ’save all.m’ is provided.

Each format has its unit test suites developed with carefully selected original data samples and manipulated versions to cover the possible use cases and failures for verification, intending to support future contributions without introducing any regression.

### Usage

Below is an example of parsing a popular library format, such as PSPLIB or MMLIB (i.e., *.mm files), into PDM variables.

*Usage*: PDM = parse xlib(’../data/mmlib100/J1001_4.mm’,1), where the first argument is the data folder and instance, and the second argument is one of the simulation trade-off problem types (for multimodal completion), i.e., 1 = NTP (no trade-off problem), 2 = CTP (continuous trade-off problem), or 3 = DTP (discrete trade-off problem).

*Output*: PDM = [DSM,TD,CD,{QD,RD}], the project domain matrix (PDM) variable containing specific domains as submatrices.

Datasets can also be exported to MAT container files with the desired format including all supported simulation types (trade-off problem).

*Usage*: save instance(’../data/mmlib100’,’xlib’), where the first argument is the data folder containing instances, and the second argument is the database format group.

*Output*: The output files are stored in the folder ../data/mmlib100_output/*.mat with all exported MAT files containing PDM variables.

All datasets within the data folder can be exported for all supported simulation types (trade-off problem) as well.

### *Usage*: save all

*Output*: The output files are stored in the folder ../data/<library>_output/*.mat with the exported MAT file containers for all supported simulation types.

To run the corresponding unit tests for each parser (library format):

*Example*: results = run(parse_xlib_test) or runtests(’parse_xlib_test’) Note: The ../test_data/ folder contains some necessary input test files for the provided unit tests.

### Method validation

The proposed parser collects project structures from seven single-project (see Table 3(a)) and five databases containing multiproject structures (see Table 3(b)). The parser converts the 15,945 single and 462,341 multiple projects and stores them in the proposed matrix-based project database.

**Table 3:** Numbers of project structures in the parsed project databases.

(a) Number of parsed project structures in single-project databases				
Database	Dataset	Structures	Avg. tasks	Reference
Boctor	Boctor	432	75.00	Boctor [3]
Kolisch	SMCP	360	29.00	Kolisch et al. [10]
	SMFF	834	30.00	
MMLIB	MMLIB50	972	50.00	Peteghem and Vanhoucke [18]
	MMLIB100	972	100.00	
	MMLIB+	5832	75.00	
Patterson	Patterson	198	24.02	Patterson [17]
PSPLIB	j30	1152	30.00	Sprecher and Kolisch [19]
	j30sm	864	30.00	
Real-life	PROTRACK	225	65.56	Batselier and Vanhoucke [2]
RG	RG30	3240	30.00	Vanhoucke et al. [22]
	RG300	864	300.00	
Sum		15,945		

(b) Number of parsed project structures in multiproject databases				

BY	BY	110,880	60.00	Browning and Yassine [4]
MPLIB1	Set 1	7497	360.00	Van Eynde and Vanhoucke [20]
	Set 2	13,167	720.00	
				
	Set 3	20,286	1440.00	
MPLIB2	Set 1	91,125	1000.00	Van Eynde and Vanhoucke [20]
	Set 2	77,760	1000.00	
	Set 3	77,760	1000.00	
	Set 4	69,12	1000.00	
MPSPLIB	MPSPLIB	1260	872.14	Homberger [9]
RCMPSPLIB	RCMPSPLIB	234	164.62	Vázquez et al. [23]
Sum		462,341		

Table 3 shows that the parsed project structures have different numbers of tasks. Kosztyan et al. [15] also showed that these projects have different structural, time, and resource-related indicators. However, these are the key factors in algorithms for scheduling and allocating resources. Comparing methods and solution approaches is of not only theoretical but also practical importance, as Vanhoucke [21] showed that projects in different fields have different structures. For example, an IT project involves many more parallel tasks than, say, an investment project. In addition, the proposed matrix-based model makes it possible to extend project plans with quality, cost, and flexibility parameters.

For studying the role of the project parameters in determining the effectiveness of scheduling approaches from a theoretical point of view, scholars obtain a research map where existing, implemented algorithms are compared, considering project parameters to decide which scheduling approach should be used. Most resource allocation problems are combinatorial, NP-hard problems. Therefore, from a practical point of view, it is important to study which approaches can be used to obtain at least one near-optimal solution as quickly as possible. By addressing the flexibility between tasks and the priorities of task completion, the original project plans can be transformed into flexible, e.g., agile, hybrid, or extreme, project plans. Today, there are still very few algorithms that can handle all the listed parameters. Nevertheless, the existing trade-off problems can be extended to consider quality, cost, and flexibility parameters.

The proposed matrix-based model addresses cost and nonrenewable demands and quality parameters and manages multiple completion modes and multilevel projects. Nevertheless, to ensure a good comparison between simulated and real-life projects, Kosztyan et al. [15] examined mainly single-project, single-mode environments with time and renewable-resource demands. The proposed parsers also collect data on multiprojects and multimodal completion modes. Therefore, comparing project parameters can also be extended to include these kinds of projects. The proposed matrix-based model and the implemented parser not only unify heterogeneous databases and eliminate the challenge of working with diverse formats but also allow the user to test both traditional and flexible project-scheduling algorithms.

The implemented parsers are freely available. They can be downloaded from MATLAB Central. The developer version can be downloaded from GitHub. The verified capsule was published on Code Ocean's site. Solvers for matrix-based project planning can also be downloaded from MATLAB Central. The software license follows the MIT license. Therefore, all software is freely available for both scholarly and commercial use.

## Conclusions

In this paper, project structure parsers are introduced, which collect the most frequently used simulated and real-life project databases and convert them to a matrix-based database. The proposed matrix-based methods enable users to add further domains and features, such as quality, cost, and flexibility parameters. The software is implemented for our original paper Kosztyan et al. [15], although it is mainly focused on individual projects and single completion modes, while the proposed parsers can process multiproject structures and multiple completion modes.

## Limitations

*Not applicable.*

## Ethics statements

*Not applicable.*

## Data availability

Data can be downloaded by using the proposed parser. It is freely available and it can be downloaded from the following websites:

• https://www.mathworks.com/matlabcentral/fileexchange/123885-matrix-based-project-dataset-parsers

• https://github.com/novakge/project-parsers

• https://codeocean.com/capsule/9344928/tree/v1

**Supplementary material *and/or* additional information [OPTIONAL]**

*Not applicable*

## CRediT authorship contribution statement

**Zsolt T. Kosztyán:** Conceptualization, Methodology, Software, Writing – original draft, Writing – review & editing. **Gergely L. Novák:** Data curation, Software, Writing – review & editing.

## Declaration of competing interest

The authors declare that they have no known competing financial interests or personal relationships that could have appeared to influence the work reported in this paper.
